# Synergistic insights: the integrated role of CT/CTP and clinical parameters in hemorrhagic transformation prediction

**DOI:** 10.18632/aging.206026

**Published:** 2024-08-09

**Authors:** Jianwen Jia, Zeping Jin, Jing Dong, Jumei Huang, Yang Wang, Yunpeng Liu

**Affiliations:** 1 Department of Neurosurgery, Beijing Chao-Yang Hospital, Capital Medical University, Beijing, People’s Republic of China; 2 Department of Medical Engineering, Tsinghua University Yuquan Hospital, Beijing, People’s Republic of China

**Keywords:** hemorrhagic transformation, ischemic stroke, Computed Tomography perfusion, prediction model, modified Rankin Scale

## Abstract

Background: Acute ischemic stroke presents significant challenges in healthcare, notably due to the risk and poor prognosis associated with hemorrhagic transformation (HT). Currently, there is a notable gap in the early clinical stage for a valid and reliable predictive model for HT.

Methods: This single-center retrospective study analyzed data from 224 patients with acute ischemic stroke due to large vessel occlusion. We collected comprehensive clinical data, CT, and CTP parameters. A predictive model for HT was developed, incorporating clinical indicators alongside imaging data, and its efficacy was evaluated using decision curve analysis and calibration curves. In addition, we have also built a free browser-based online calculator based on this model for HT prediction.

Results: The study identified atrial fibrillation and hypertension as significant risk factors for HT. Patients with HT showed more extensive initial ischemic damage and a smaller ischemic penumbra. Our novel predictive model, integrating clinical indicators with CT and CTP parameters, demonstrated superior predictive value compared to models based solely on clinical indicators.

Conclusions: The research highlighted the intricate interplay of clinical and imaging parameters in HT post-thrombectomy. It established a multifaceted predictive model, enhancing the understanding and management of acute ischemic stroke. Future studies should focus on validating this model in broader cohorts, further investigating the causal relationships, and exploring the nuanced effects of these parameters on patient outcomes post-stroke.

## INTRODUCTION

Stroke represents a significant global health burden, accounting for a substantial number of disabilities and deaths worldwide. Since 2015, stroke has become the leading cause of death and disability in China, posing a serious threat to national health. With the rapid aging of China’s population, the number of stroke patients is expected to increase dramatically. And acute ischemic stroke (AIS) patients account for more than 80% of stroke patients. Therefore, it is urgent to pay attention to AIS patients [1, 2]. The rapid advancement of endovascular procedures, particularly thrombectomy, has revolutionized the management of AIS, offering hope for improved outcomes [3, 4]. However, the success of these interventions is not without challenges, as they are associated with various complications, including hemorrhagic transformation (HT) [5].

HT is a frequent and serious complication following thrombectomy, often leading to a worsening prognosis, underscoring the need for a deeper understanding of its pathophysiology and predictors [6, 7]. Despite its clinical significance, the exact cause of HT in the context of post-thrombectomy care remains elusive. This lack of clarity complicates the management of patients undergoing thrombectomy and highlights the necessity for more targeted therapeutic strategies.

The complexity of acute ischemic stroke, coupled with the individual variability in patient response to thrombectomy, suggests that a multifactorial approach is required to predict the likelihood of HT. In this regard, a comprehensive analysis of patients’ baseline characteristics, including clinical and demographic data, in conjunction with detailed radiologic findings, particularly from cerebral perfusion imaging like Computed Tomography perfusion (CTP), emerges as a promising strategy. Such an approach could potentially identify patients at higher risk for HT, allowing for more personalized and effective management strategies.

The integration of clinical and radiological data to predict HT is not only a step forward in personalized medicine but also a stride towards improving the overall outcomes in acute ischemic stroke management. By identifying high-risk individuals, clinicians can tailor their approach, potentially mitigating the risk of HT and its associated complications. This study aims to explore these correlations and contribute to the evolving landscape of stroke care.

## RESULTS

### Descriptive clinical characteristics of subjects

From January 2021 to August 2023, our department managed a total of 224 patients suffering from large vessel-occlusion acute ischemic stroke. These patients were categorized based on the outcomes following thrombectomy: 175 patients were identified with No HT, while 49 patients experienced HT as observed in post-thrombectomy cranial CT scans within 7 days. There was no significant difference in the average age and gender distribution among these groups.

In analyzing the comorbidity profiles of these patients, we observed distinct patterns between the two groups. In the HT group, 36.7% of patients had atrial fibrillation, compared to only 17.1% in the No HT group, a finding that was statistically significant. However, for other comorbidities such as hypertension, diabetes, coronary artery disease, and smoking history, there were no significant differences observed between the HT and No HT groups. Additionally, we collected data on the systolic blood pressure and serum glucose levels of patients upon admission to our hospital. Analysis of these parameters showed no significant differences between the groups.

Significant differences were noted in the National Institutes of Health Stroke Scale (NIHSS) scores on admission between the two groups. Patients in the No HT group had lower NIHSS scores, with a median of 11 (interquartile range (IQR), 7 to 15), compared to those with HT, who had higher scores, with a median of 13 (IQR, 10 to 16). Additionally, a higher proportion of patients in the HT group received intravenous thrombolysis with recombinant tissue plasminogen activator (rt-PA) before undergoing thrombectomy, at 11.6%, compared to only 3.6% in the No HT group, which was statistically significant. Furthermore, the Door-to-Puncture Time (DPT) in patients with HT (median 82.5 minutes, IQR 74.8 to 101.5) was significantly shorter than that in the No HT group (median 93.0 minutes, IQR 83.0 to 116.5). Despite these differences, the Time from Stroke Onset to Groin Puncture was similar between the two groups, indicating no significant variation in the overall treatment initiation timeframe. The above clinical information was illustrated in Table 1.

**Table 1:** Descriptive clinical characteristics of subjects.

	**No HT (*n* = 175)**	**HT (*n* = 49)**	***P*-Value**
Age	66.3 ± 10.9	69.0 ± 11.5	*P* = 0.1266
Gender (Male)	128 (73.6%)	33 (67.4%)	*P* = 0.3909
**Co-Morbidity**
Atrial Fibrillation	30 (17.1%)	18 (36.7%)	***P* = 0.0061**
Hypertension	115 (65.7%)	37 (75.5%)	*P* = 0.2697
Diabetes	54 (30.9%)	10 (20.4%)	*P* = 0.1334
Coronary Artery Disease	30 (17.1%)	10 (20.4%)	*P* = 0.2743
Smoking	68 (38.9%)	16 (32.7%)	*P* = 0.4278
Systolic Blood Pressure on Admission (mmHg)	149.8 ± 25.2	144.9 ± 26.8	*P* = 0.2341
Serum Glucose Level on Admission (mmol/L)	6.8 (6.0, 8.3)	7.3 (6.3, 8.3)	*P* = 0.3908
NIHSS on Admission	11 (7, 15)	13 (10, 16)	***P* = 0.0034**
Bridging Thrombectomy	5 (3.6%)	5 (11.6%)	***P* = 0.0407**
DPT (min)	93.0 (83.0, 116.5)	82.5 (74.8, 101.5)	***P* = 0.0035**
Time from Stroke Onset to Groin Puncture (min)	300.0 (202.5, 480.0)	360.0 (240.0, 480.0)	*P* = 0.1567

Abbreviations: DPT: Door-to-Puncture Time; No HT: non-hemorrhagic transformation; HT: hemorrhagic transformation. The Chi-Square test was employed to analyze nonparametric data. The Student’s *t*-test was used for parametric data.

### CTP findings and perfusion deficits in HT patients

**Figure 1:**
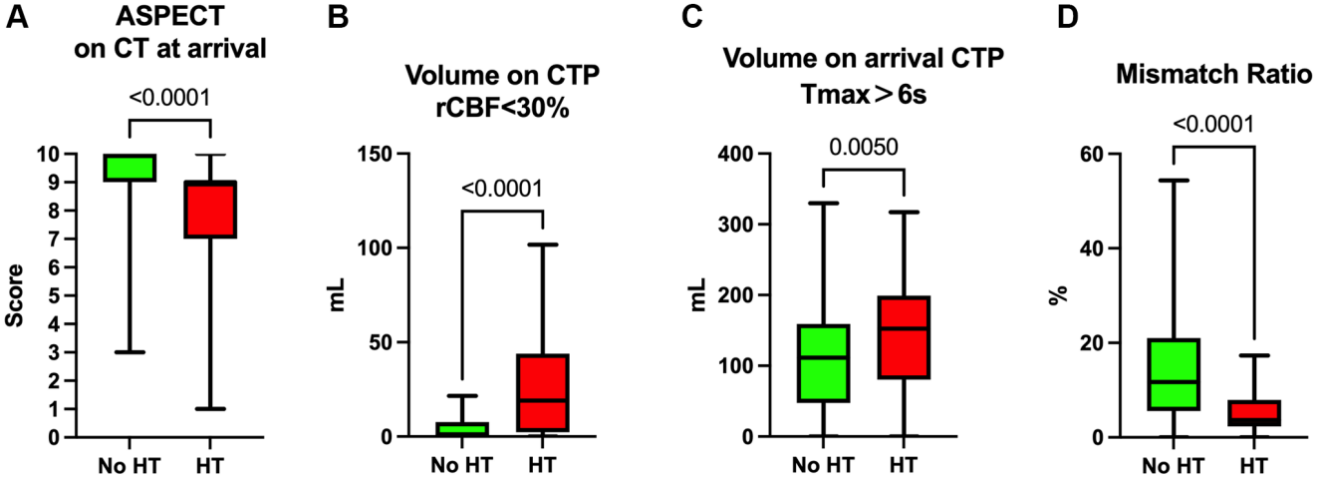
**CT and CTP analysis of patients on admission.** (**A**) ASPECT of CT results on admission of the patients. (**B**) Volume of rCBF<30% on admission CTP. (**C**) Volume of Tmax>6s on admission CTP. (**D**) Mismatch ratio (volume of Tmax>6s/volume of rCBF<30%).

Upon admission, computed tomography (CT) and perfusion CT (CTP) imaging were performed on our patients to assess the extent of ischemic stroke. The Alberta Stroke Program Early CT Score (ASPECTS) was utilized to evaluate initial brain ischemia. Patients with no HT demonstrated significantly higher ASPECTS, with a median score of 10 (IQR, 9 to 10), as opposed to those with HT who had lower scores with a median of 9 (IQR, 7 to 9) (Figure 1A).

For CTP imaging metrics, the volume of regions with reduced cerebral blood flow (rCBF) less than 30%, which reflects the core infarction zone, was considerably larger in the HT group with a median volume of 19.1 mL (IQR, 2.3 to 43.9 mL), compared to the No HT group, which had a median volume of 0.9 mL (IQR, 0.0 to 7.7 mL) (Figure 1B). Moreover, the volume of brain tissue experiencing a time-to-maximum (Tmax) greater than 6 seconds was significantly larger in the HT group (median 152.6 mL, IQR 80.4 to 199.1 mL) versus the No HT group (median 111.4 mL, IQR 47.5 to 159.3 mL), suggesting more extensive brain tissue at risk of progressing to infarction (Figure 1C).

Additionally, the mismatch ratio, indicative of the ischemic penumbra—the area of salvageable tissue surrounding the core infarct—was significantly smaller in the HT group (median 3.7, IQR 2.3 to 7.9) when compared to the No HT group (median 11.7, IQR 5.6 to 21.0). This suggests a more limited therapeutic window and potentially lower benefit from reperfusion therapy in the HT group. These imaging findings highlight the value of early and precise CTP assessment in guiding clinical decisions for acute stroke interventions (Figure 1D).

### Patients with HT had longer recanalization time during thrombectomy

In the evaluation of endovascular thrombectomy outcomes, recanalization success was measured using the modified Thrombolysis in Cerebral Infarction (mTICI) scale. The proportion of patients achieving mTICI 2b-3 recanalization, indicative of substantial reperfusion, did not differ significantly between the groups, with 85.6% in the No HT group and 87.8% in the HT group. Similarly, the rate of achieving one-pass recanalization showed no significant difference between the No HT group (47.3%) and the HT group (40.8%) (Table 2).

**Table 2:** mTICI scale and the one-pass recanalization of patients.

	**No HT (*n* = 175)**	**HT (*n* = 49)**	***P*-Value**
mTICI (2b-3)	149 (85.6%)	43 (87.8%)	*P* = 0.7044
One-Pass Recanalization	80 (47.3%)	20 (40.8%)	*P* = 0.4199

Abbreviations: mTICI: modified Thrombolysis in Cerebral Infarction; No HT: non-hemorrhagic transformation; HT: hemorrhagic transformation. The Chi-Square test was employed to analyze nonparametric data.

**Figure 2:**
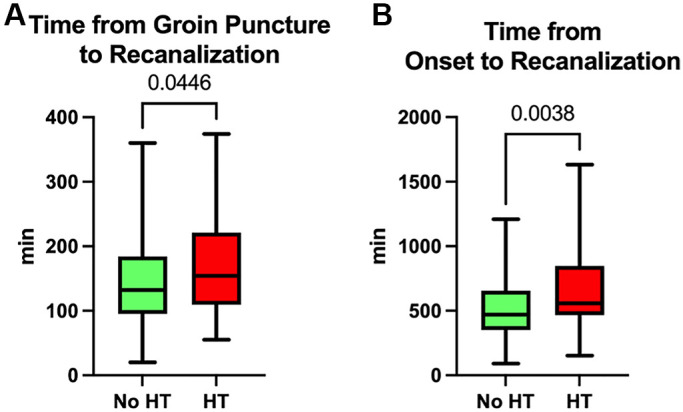
**Time spent during thrombectomy.** (**A**) Time from groin puncture to recanalization. (**B**) Time from stroke onset to recanalization.

However, the duration from groin puncture to recanalization was notably longer in the HT group, with a median time of 154 minutes (interquartile range (IQR), 109.5 to 221.0 minutes), compared to the No HT group, which had a median time of 132.0 minutes (IQR, 95.0 to 184.0 minutes) (Figure 2A). Moreover, when considering the total elapsed time from stroke onset to recanalization, patients in the HT group experienced a longer duration with a median time of 557.0 minutes (IQR, 466.5 to 847.5 minutes), as opposed to the No HT group, which had a median time of 470.5 minutes (IQR, 351.5 to 655.5 minutes) (Figure 2B).

### Independent risk factors for HT in IS

**Figure 3:**
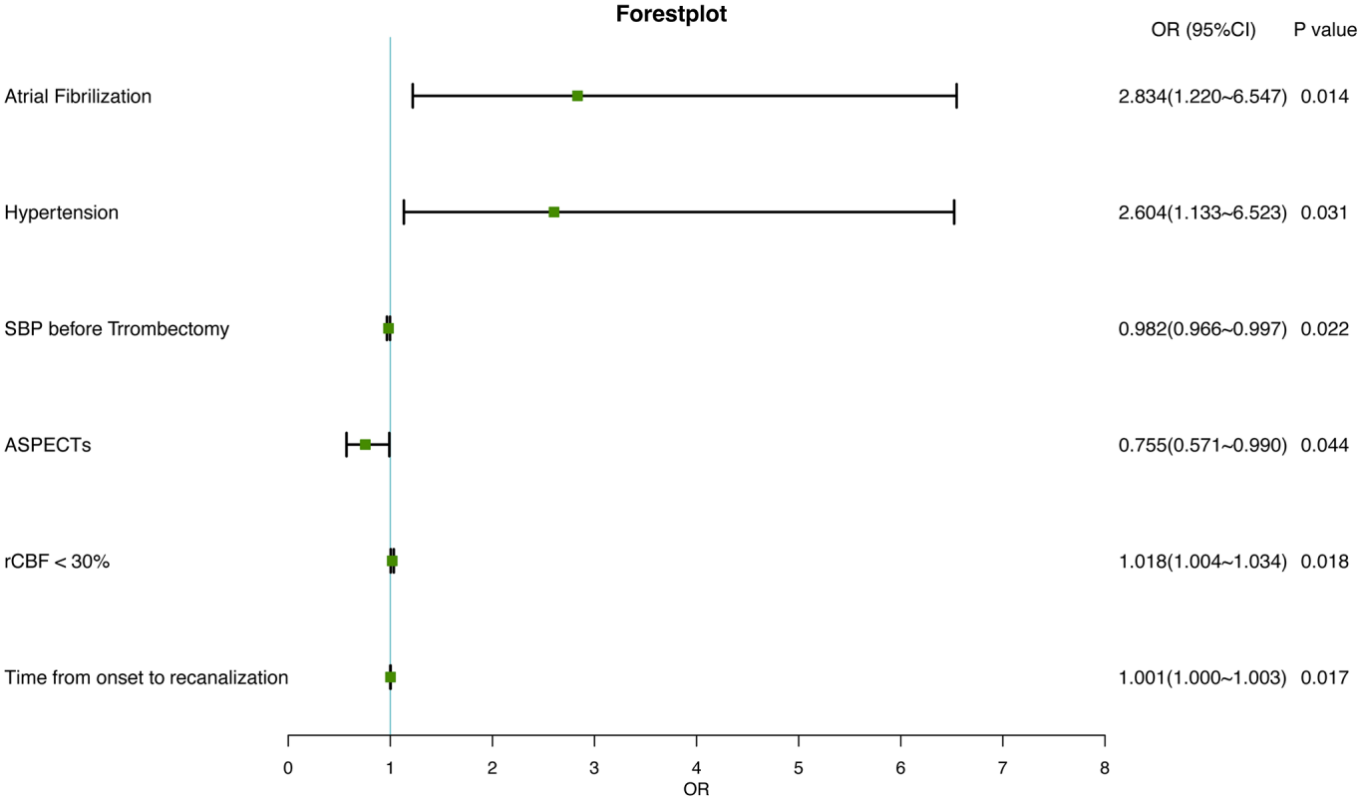
Forest plot of independently associated factors for HT in IS.

The results of univariate and multivariate logistic analyses between patients with HT and those without HT are presented in Table 3. The univariate analysis suggests that compared to patients without HT, the patients with HT are more likely to have AF history (OR, 2.863; 95% CI, 1.404–5.785; *P* = 0.003), to have higher NIHSS scores (OR, 1.038; 95% CI, 0.997–1.081; *P* = 0.068) and ASPECTS (OR, 0.631; 95% CI, 0.506–0.770; *P* < 0.001), to have a larger volume of regions with rCBF less than 30% (OR, 1.026; 95% CI, 1.015–1.039; *P* < 0.001), to have a larger volume of brain tissue experiencing Tmax greater than 6 seconds (OR, 1.006; 95% CI, 1.001–1.010; *P* = 0.009), and to have a longer time from stroke onset to recanalization of the offending vessel (OR, 1.001; 95% CI, 1.000–1.002; *P* = 0.025). Factors with a *p*-value less than 0.1 in the univariate analysis, along with factors previously proven to be potentially related to HT in other studies, were included in the multivariate logistic regression analysis. After optimizing the model fitting through stepwise forward and backward methods and adjusting for other factors, a history of AF (OR, 2.834; 95% CI, 1.220–6.547; *P* = 0.014) and hypertension (OR, 2.604; 95% CI, 1.133–6.523; *P* = 0.031), lower preoperative blood pressure (OR, 0.982; 95% CI, 0.966–0.997; *P* = 0.022), lower ASPECTS (OR, 0.755; 95% CI, 0.571–0.990; *P* = 0.044), a larger volume of brain tissue with rCBF less than 30% (OR, 1.018; 95% CI, 1.004–1.034; *P* = 0.018), and a longer time from symptom onset to recanalization (OR, 1.001; 95% CI, 1.000–1.003; *P* = 0.017) were identified as independent risk factors for HT (Table 3, Figure 3).

**Table 3:** Univariate and multivariate logistic regression analysis.

**Character**	**Unadjusted**			**Adjusted**		
Age	1.024	0.994–1.055	0.124			
Gender (Male)	0.776	0.395–1.565	0.467			
Co-Morbidity						
Atrial Fibrillation	2.863	1.404–5.785	0.003	2.834	1.220–6.547	0.014
Hypertension	1.652	0.821–3.521	0.173	2.604	1.133–6.523	0.031
Diabetes	0.560	0.249–1.167	0.138			
Coronary Artery Disease	1.056	0.568–2.876	0.674			
Smoking	0.779	0.390–1.506	0.465			
Systolic Blood Pressure on Admission (mmHg)	0.992	0.979–1.004	0.194	0.982	0.966–0.977	0.022
Serum Glucose Level on Admission (mmol/L)	0.920	0.812–1.023	0.155			
NIHSS on Admission	1.038	0.997–1.081	0.068			
Bridging Thrombectomy	0.718	0.255–1.743	0.491			
DPT (min)	0.992	0.981–1.001	0.126			
Time from Stroke Onset to Groin Puncture (min)	1.001	1.000–1.002	0.202			
Alberta Stroke Program Early CT Score	0.631	0.506–0.770	<0.001	0.755	0.571–0.990	0.044
rCBF<30%	1.026	1.015–1.039	<0.001	1.018	1.004–1.034	0.018
Tmax>6s	1.006	1.001–1.010	0.009			
Time from stroke onset to recanalization (min)	1.001	1.000–1.002	0.025	1.001	1.000–1.003	0.017

Abbreviations: NIHSS: National Institutes of Health Stroke Scale; DPT: Door-to-Puncture Time; rCBF: relative cerebral blood flow; Tmax: time-to-maximum.

### Development and evaluation of an HT-predicting nomogram

**Figure 4:**
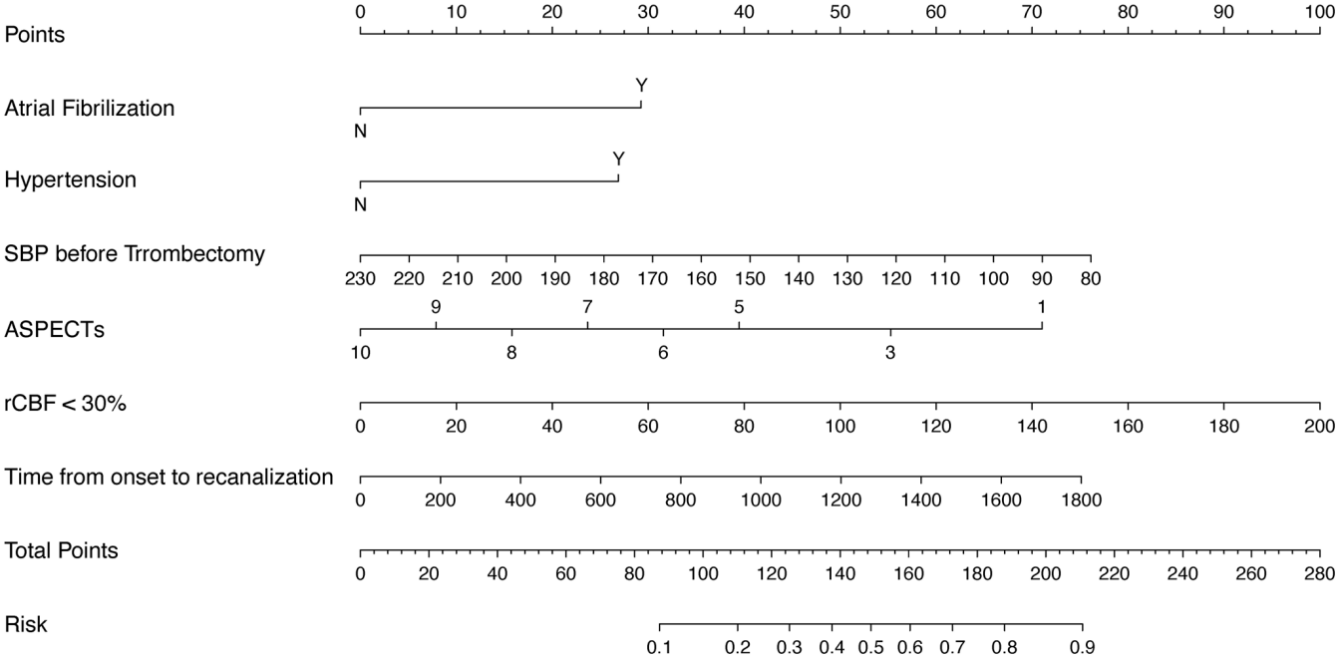
Nomogram predicting HT risk in IS patients.

**Figure 5:**
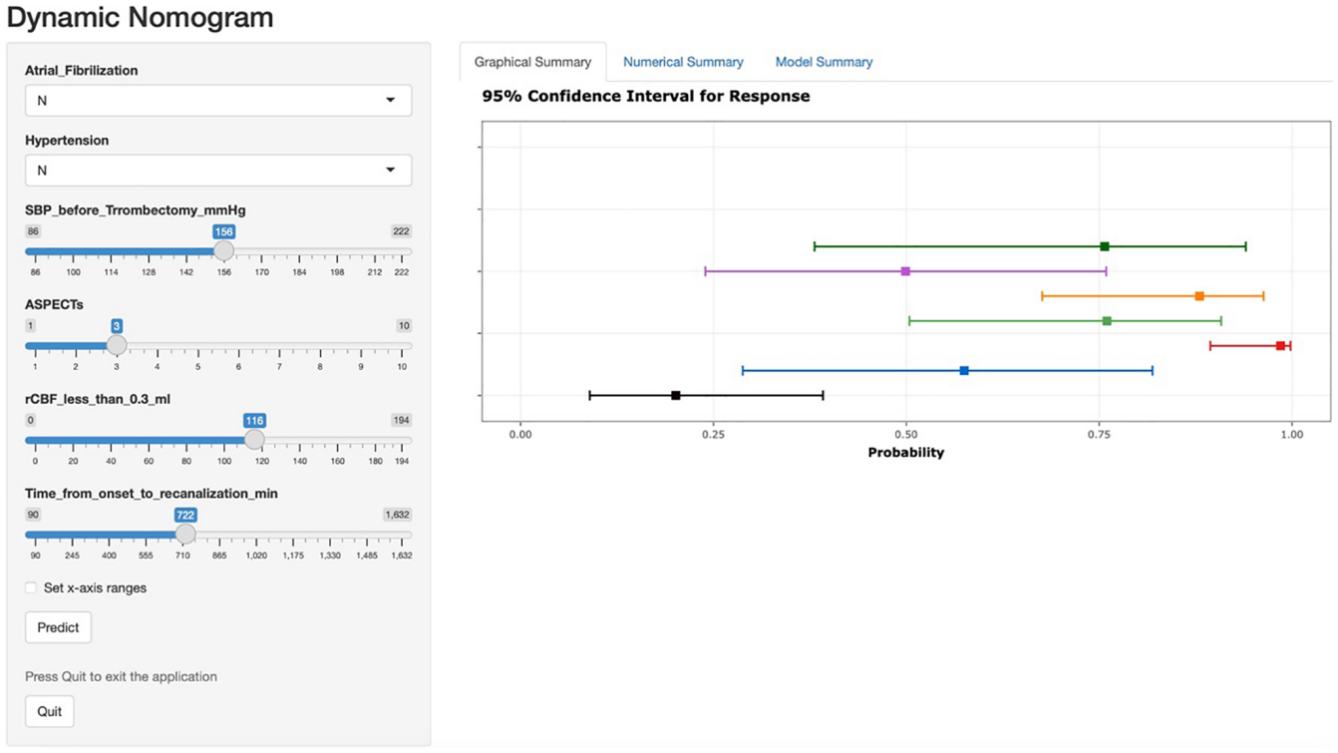
**A free browser-based online calculator based on nomogram predicting HT.** The left side of the figure shows the relevant risk factor options. Fill in the patient’s individual status according to the options, such as the presence of atrial fibrillation, the presence of hypertension, the value of preoperative systolic blood pressure, and so on. The probability of postoperative HT for the individual patient is generated after filling in the patient’s individual risk factor status, and the probability and 95% confidence intervals for response are shown on the right.

**Figure 6:**
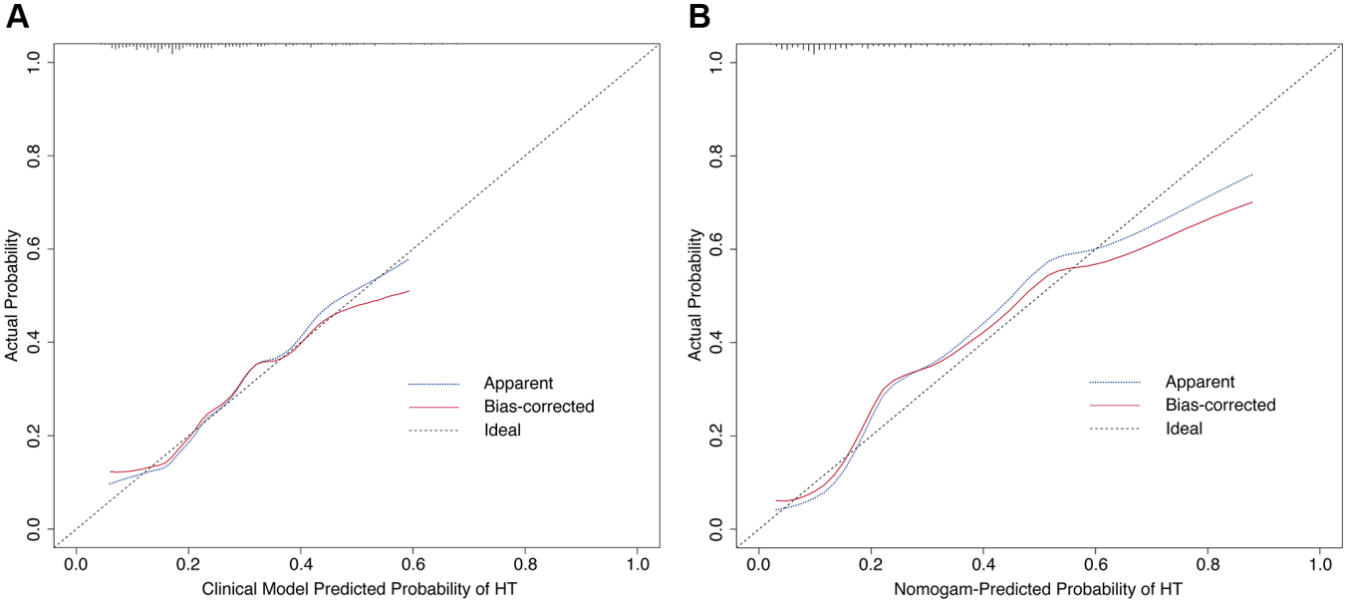
**Calibration curves of the prediction model.** (**A**) Calibration curve based on clinical indicators. (**B**) Calibration curve based on nomogram’s calculation model.

**Figure 7:**
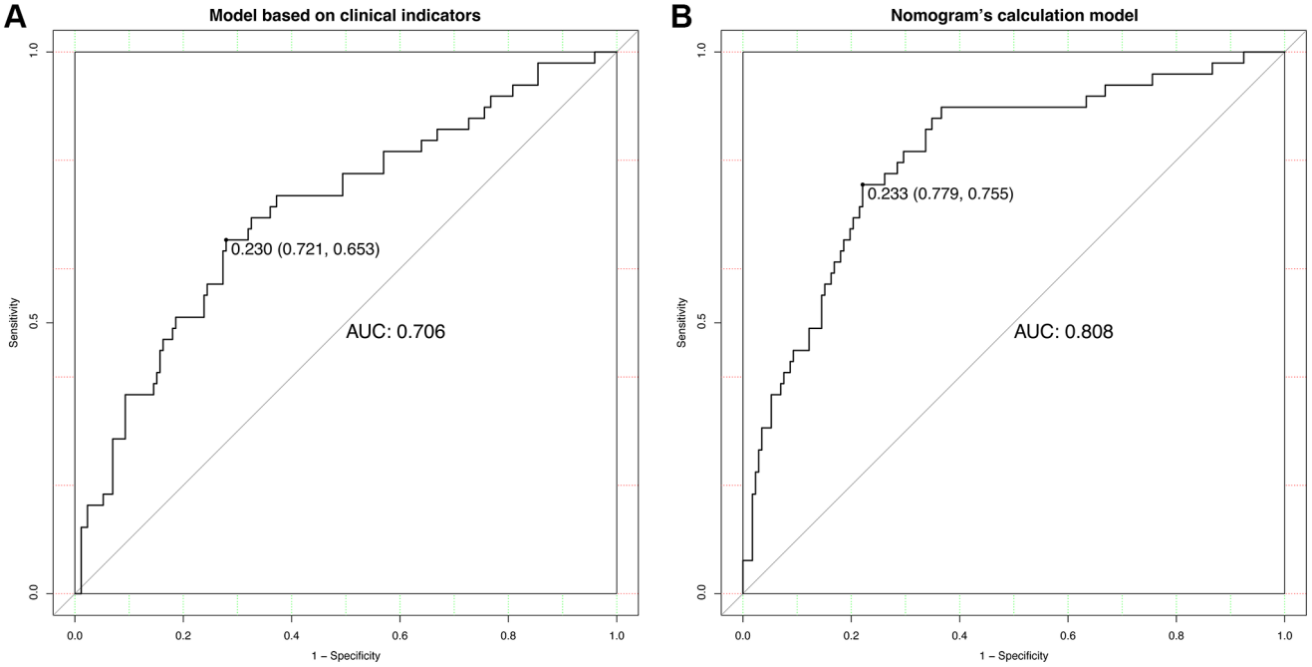
**ROC curve analyses.** (**A**) Analysis of the model based on clinical indicators. (**B**) Analysis based on nomogram’s calculation model.

**Figure 8:**
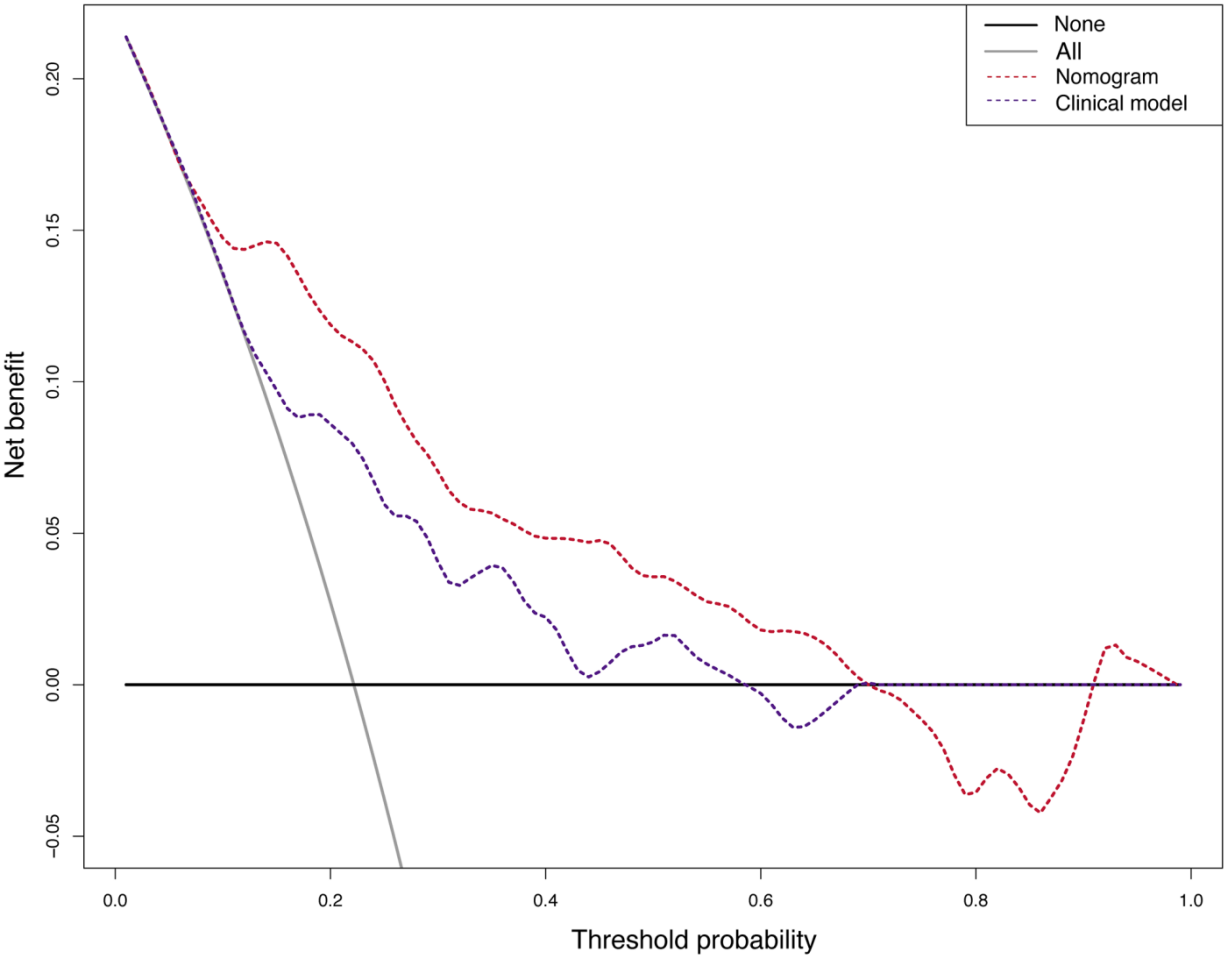
The decision curve analysis (DCA) of the nomogram’s calculation model and the model based on clinical indicators.

A nomogram for predicting HT was established based on an equation derived from these independent risk factors (Figure 4). The score of each independent predictor corresponds to the score on the upper scale, and the total score for each subject is the sum of the scores from each independent predictor. The total score corresponds to the risk of HT on the risk axis, with higher total scores indicating a higher risk of HT. The nomogram we constructed is available through a free browser-based online calculator at https://chao-yang-hospital-neurosurgery.shinyapps.io/Nomogram_predicting_HT/ (Figure 5). The calibration curve (Figure 6) suggests that, compared to the model based solely on clinical indicators, the nomogram’s calculation model incorporating CT and CTP indicators shows overall better coherence between the predicted probability of HT and the actual clinical characteristics of HT. Additionally, the Receiver Operating Characteristic (ROC) curve (Figure 7) of the model incorporating CT and CTP indicators also performs better. The AUC for the model built on clinical indicators is 0.706. In contrast, the ROC curve for the nomogram’s calculation model that includes clinical and radiological indicators has a larger AUC of 0.808, with the model’s sensitivity and specificity being 0.755 and 0.779, respectively. Decision curve analysis (DCA) curves demonstrate that the nomogram is more effective in predicting HT, as it provides greater net benefits for identifying HT in IS patients across the threshold probabilities from 0.2 to 0.7, compared to predictions based solely on clinical indicators and with both the intervention for all and the intervention for none (Figure 8).

### Patients with HT had higher mRS 90 days after onset of stroke

**Figure 9:**
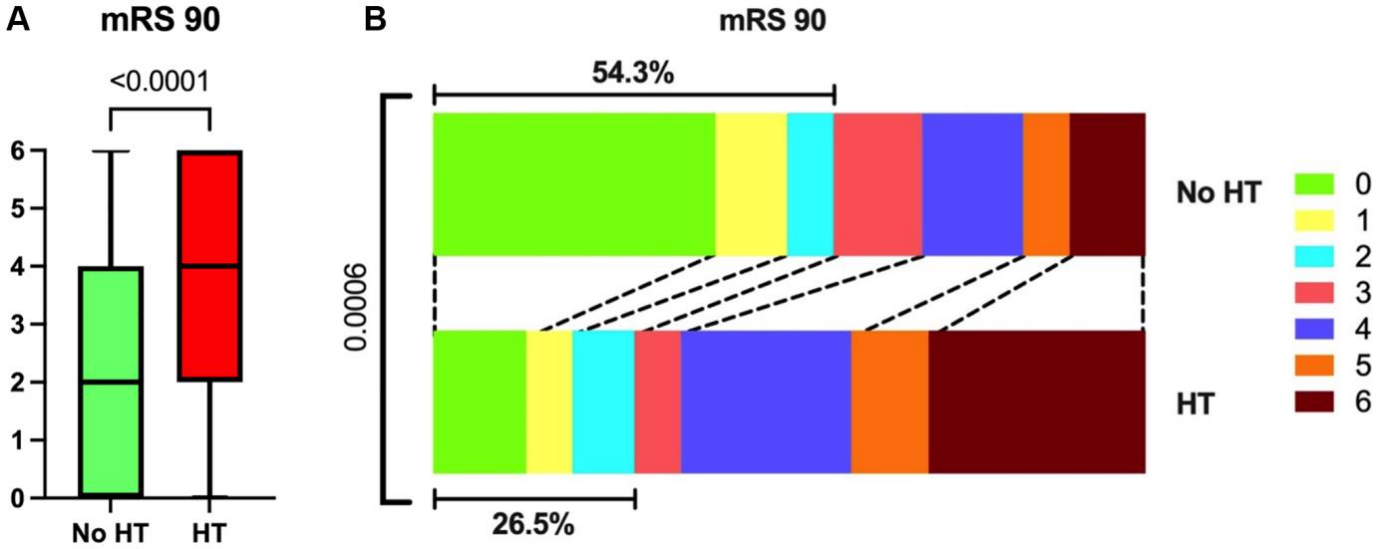
**mRS 90 of all participants.** (**A**) Patients in the HT group had higher mRS 90 than those in the No HT group. (**B**) The proportion of patients who reached 0–2 on mRS 90 was lower in the HT group.

The prognosis of patients post-thrombectomy was quantitatively assessed using the modified Rankin Scale (mRS) at 90 days following the onset of stroke. Our findings indicated that patients in the HT group had a higher median 90-day mRS score of 4 (IQR: 2 to 6), suggesting greater disability, compared to the No HT group, which had a median 90-day mRS score of 2 (IQR: 0 to 4), (Figure 9A). Furthermore, the percentage of patients achieving a favorable functional outcome, defined as a 90-day mRS score of 0 to 2, was significantly higher in the No HT group (54.3%) compared to the HT group (26.5%) (Figure 9B).

## DISCUSSION

Our study, encompassing a cohort of 224 patients with large vessel-occlusion acute ischemic stroke (AIS-LVO), discovered significant differences in co-morbidities, particularly atrial fibrillation, which was more prevalent in patients who developed HT post-thrombectomy. Imaging analysis revealed that HT patients had more extensive initial ischemic damage and a smaller ischemic penumbra. Despite achieving similar rates of successful recanalization, the HT group had longer recanalization times and poorer 90-day functional outcomes as measured by the mRS. These findings point to the complexity of HT development and its implications for post-thrombectomy care.

The incidence of HT post-endovascular thrombectomy (EVT) was notably higher among patients with AF, reflecting the condition’s severity due to the larger clot burdens typically associated with cardiac embolism. This leads to a rapid progression of ischemic damage and limited opportunity for collateral circulation development. In contrast, strokes from cerebral atherosclerosis typically evolve more gradually, allowing for some compensatory circulatory mechanisms to form. The presence of nonsustained atrial tachycardia (NSAT) in ischemic stroke patients, which is associated with a higher proportion of embolic strokes, further suggests that AF and cardiac embolism significantly impact the severity of strokes [8]. Moreover, atrial fibrillation is responsible for a significant percentage of ischemic strokes and is linked with increased stroke severity and complications, as highlighted in the research by Zhou et al. [9]. These differences underscore the importance of adopting individualized stroke treatment strategies, which thoroughly consider the distinct etiological factors and their respective impact on therapeutic outcomes and the likelihood of HT.

Additionally, our findings underscore hypertension as another pivotal risk factor for HT. Corresponding with literature that has documented hypertension’s contribution to increased stroke severity and hemorrhagic complications [10], our study echoes these observations. Notably, the relationship between blood pressure and HT is intricate, as evidenced by our data showing a negative correlation between systolic blood pressure on admission and the occurrence of HT. A recent study also suggests that aggressively lowering blood pressure after a thrombectomy might not be beneficial and could even harm recovery [11]. These insights point to the need for a balanced approach to managing blood pressure after stroke, tailored to each patient’s situation. Further research is crucial to fully understand these dynamics and improve stroke care.

Our study’s imaging analysis indicated that patients with hemorrhagic transformation (HT) post-thrombectomy had more extensive initial ischemic damage and a smaller ischemic penumbra, suggesting a higher risk of hemorrhage upon reperfusion due to less salvageable brain tissue. This finding is consistent with Shao et al.’s research, which demonstrates a strong correlation between postinterventional cerebral hyperdensity after mechanical thrombectomy and HT in acute ischemic stroke patients [12]. Furthermore, despite similar success rates in recanalization, the HT group exhibited longer recanalization times and poorer 90-day functional outcomes, as measured by the mRS. This complexity in HT development is underscored by Van Kranendonk et al., who reported an association between larger hemorrhage volumes, particularly in parenchymal hematomas, and functional outcomes, highlighting the prognostic value of hemorrhage size in stroke patients [13].

Moreover, studies have demonstrated that CT perfusion parameters, including cerebral blood flow and volume, can predict salvageable ischemic penumbra and the risk of HT after mechanical thrombectomy. Kameda et al. established optimal thresholds for detecting ischemic penumbra using CT perfusion, highlighting its potential in guiding treatment decisions for stroke patients [14]. Furthermore, Ozkul-Wermester et al. found that increased blood-brain barrier permeability on perfusion CT is predictive of HT in stroke patients, with factors like atrial fibrillation, infarct volume, and collateral status being associated with HT development [15]. These findings underscore the value of advanced radiologic techniques in acute ischemic stroke management, particularly for identifying patients at risk for HT and tailoring therapeutic strategies accordingly.

Patients with HT following EVT exhibit a poorer prognosis compared to those without HT. Elevated intracranial pressure (ICP) due to HT is a primary concern, as it can significantly impair cerebral perfusion and lead to additional ischemic injury. In some instances, to manage the increased ICP, surgical interventions such as craniotomies may be necessary to relieve pressure or evacuate the hematoma. These procedures, while potentially life-saving, carry inherent risks including infection and rebleeding, and could induce further neurological deficits due to their invasive nature [16]. Additionally, HT is associated with an amplified inflammatory response in the brain. This inflammation can worsen neuronal damage beyond what is caused by the initial ischemic stroke. Specific inflammatory biomarkers, like the counts of lymphocytes and neutrophils, have been linked to hemorrhagic complications following EVT. Such postoperative inflammation can add to the complexity of patient recovery and influence long-term outcomes [17, 18]. The challenging nature of these interactions provides ample opportunity for future research to identify more effective strategies to mitigate these risks. Advances in perioperative care and a deeper understanding of post-stroke inflammatory processes hold promise for enhancing recovery trajectories and improving outcomes for patients afflicted with HT after EVT.

In the pursuit of enhanced predictive precision, our study presents a sophisticated nomogram that incorporates CT and CTP parameters, marking a significant improvement over models based solely on clinical indicators. This refined approach not only aligns with the decision curve analysis, emphasizing its clinical utility, but also resonates with the calibration curves analysis, affirming the model’s accuracy in predicting hemorrhagic transformation post-thrombectomy. These analyses collectively underscore the nomogram’s potential in advancing personalized patient care in the realm of acute ischemic stroke management.

While our study sheds light on important aspects of hemorrhagic transformation post-thrombectomy, it’s important to recognize its limitations. Being a retrospective study conducted at a single center and focused on the Chinese population, the findings might not be widely applicable. The prognostic model we’ve proposed needs validation in a larger, more varied cohort to confirm its effectiveness. Furthermore, the study didn’t include an analysis of detailed parameters like the occluded segment of the cerebral artery, which could provide deeper insights into stroke outcomes. In addition, our prediction model only predicts for patients with acute ischemic stroke and has limited applicability. In subsequent studies, we will include patients with other diseases, such as hemorrhagic stroke, craniocerebral injury, etc. We will also collect clinical and imaging data. Afterwards, we attempt to construct a comprehensive and relevant model for predicting hemorrhagic transformation applicable to multiple diseases based on these imaging and clinical data. Finally, this study did not address peripheral blood bioindicators. This was mainly limited by the sample size of this study. Therefore, in future studies, the addition of blood biomarkers after expanding the sample size may lead to an increase in the predictive efficacy of the model.

## METHODS

### Patients

This retrospective study included a total of 224 patients who underwent thrombectomy at Beijing Chao-Yang Hospital, Capital Medical University, from September 2021 to August 2023. All patients provided informed consent for their inclusion in the study. The research protocol was reviewed and approved by the Ethics Committee of Capital Medical University, ensuring adherence to ethical standards in patient treatment and data handling (2022-ke-268).

**Figure 10:**
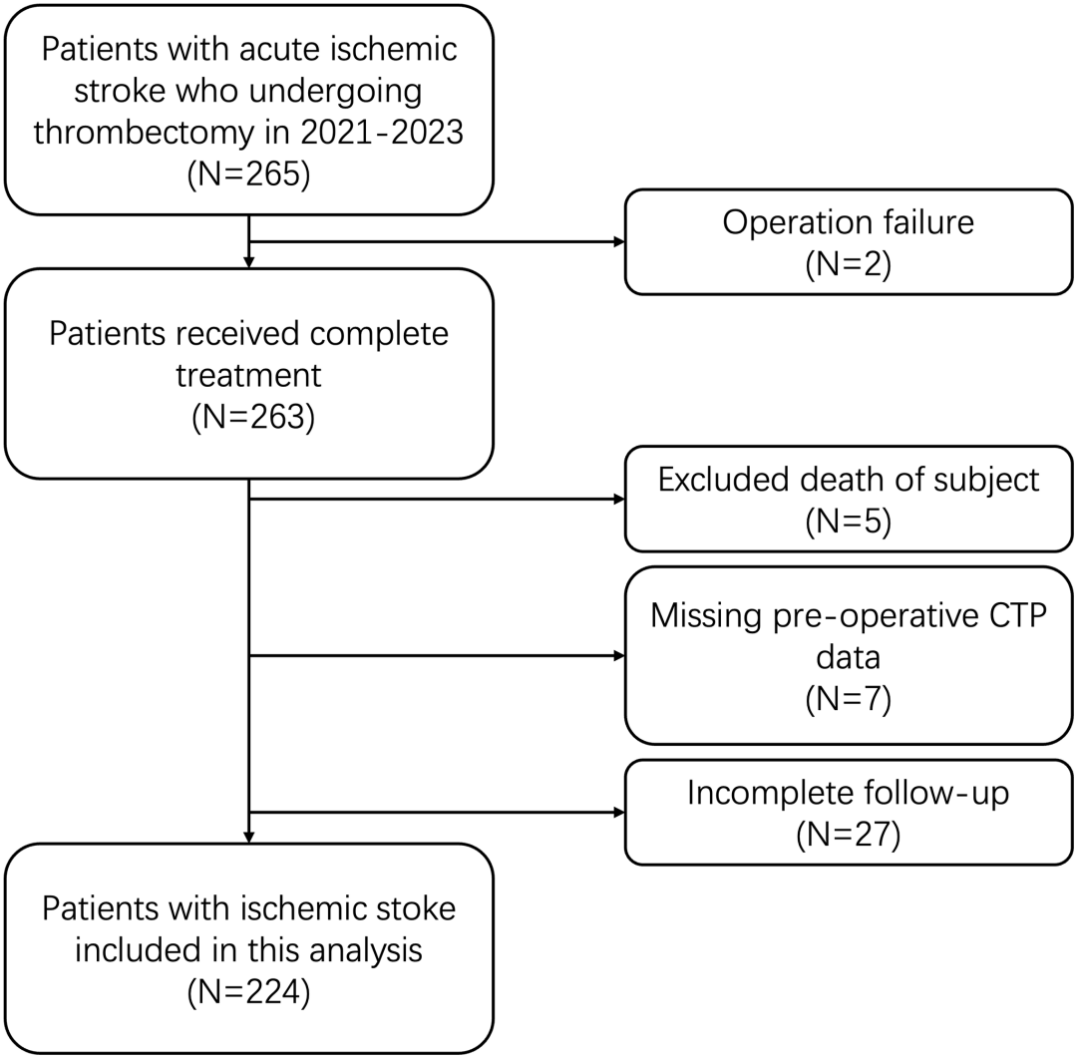
Study flow diagram.

We screened potential patients according to strict inclusion and exclusion criteria. The inclusion criteria for this study were: 1) age above 18 years old, 2) a confirmed diagnosis of ischemic stroke via imaging scans (computed tomography, magnetic resonance imaging) or cerebral angiography, and 3) treatment with thrombectomy at our center between September 2021 and August 2023. Exclusion criteria included: 1) failed surgery, 2) death prior to operation, 3) missing pre-operative CTP data, and 4) missing follow-up data. Ultimately, a cohort of 224 patients was included in the present study, as depicted in the study flow diagram (Figure 10).

### Diagnosis of hemorrhagic transformation

Our protocol for identifying HT involved a rigorous analysis of Computed Tomography (CT) scans performed within 7 days following thrombectomy. To ensure the reliability of the diagnosis, the imaging data were independently reviewed by three experienced radiologists. The determination of HT was based on distinct radiological evidence of hemorrhage in the brain region corresponding to the area of initial ischemic stroke. The consensus among the radiologists was necessary to confirm the presence of HT, with any discrepancies resolved through a joint review process. This structured approach to diagnosis allowed for a consistent and objective assessment of hemorrhagic transformation across our patient cohort.

### Clinical data collection

Clinical data were meticulously collected for each patient. This included demographic information (age and gender), vital signs at admission (blood pressure and blood glucose level), neurological status as assessed by the National Institutes of Health Stroke Scale (NIHSS) on admission, door to puncture time (DPT), and post-operation mTICI score determined via digital subtraction angiography (DSA). Additionally, we recorded the presence of comorbidities such as hypertension, diabetes, atrial fibrillation, coronary artery disease, and smoking history. Information on whether patients received intravenous thrombolysis prior to thrombectomy was also noted. The functional outcome was assessed using the 90-day modified Rankin Scale (mRS 90).

### CTP parameters collection

The original CTP images were automatically processed using the SHUKUN AI perfusion software (SK-CTPDoc, StrokePro V2.0, SHUKUN Technology, Beijing, China) and six parameter maps of tMIP, CBF (cerebral blood flow), CBV (cerebral blood volume), MTT (mean transit time), TTP (time to peak), and Tmax (time-to-maximum) were obtained rapidly. The infarct core (relative cerebral blood flow, rCBF<30%), ischemic area volume (Tmax>6 s) and mismatch ratio were calculated automatically. Further, the volumes of Tmax>4 s, Tmax>6 s, Tmax>8 s, Tmax>10 s, and HIR (Tmax>10 s/Tmax>6 s) were also calculated automatically. Artery input and venous outflow and threshold were automatically selected using perfusion analysis software. Time–density curve (TDC) was observed and selection of arterial input and venous outflow was manually adjusted when an abnormal time-density curve was found due to incorrect selection of arteries and veins. The key parameters collected included the volume of Tmax>6s and the volume of rCBF<30%, along with the mismatch ratio (Volume of Tmax>6s/Volume of rCBF<30%). Further details of the CTP protocol will be provided subsequently.

### Definition of hemorrhagic transformation

Hemorrhagic transformation was identified and classified based on the criteria set forth by the European Cooperative Acute Stroke Study (ECASS) [19]. This was done through post-thrombectomy CT scans, allowing for a standardized assessment of HT across all patients.

### Predictive model

Using univariate logistic regression analysis, we investigated potential predictive factors for HT and non-hemorrhagic transformation (No HT) separately. Subsequently, a multivariate logistic regression model analysis was conducted to identify factors independently associated with HT and No HT. Based on the results of the multivariate logistic regression analysis, nomograms were constructed using the rms package in R software (version 4.2.1). The nomogram was derived by scaling each regression coefficient in the multivariate logistic regression to a scale of 0–100. Using variables independently associated, a total score was calculated and then transformed into predicted probabilities. An interactive web-based dynamic nomogram application was built with Shiny, version 1.8.0. To assess the accuracy of the nomogram, internal validation of the nomogram was conducted using the bootstrap method with 1000 resamples, subsequently obtaining the calibration curve. The predictive ability of the nomogram’s calculation model for HT in IS patients was evaluated using Receiver Operating Characteristic (ROC) curves. The optimal cutoff value was determined as the point on the curve where the difference between sensitivity and 1-specificity was maximal at each horizontal coordinate point [20]. Further confirmation of the clinical effectiveness of the nomogram was carried out through Decision Curve Analysis (DCA), a method for assessing the clinical utility of predictive models [21]. The nomogram was evaluated by calculating the net benefit at different threshold probabilities. DCA, capable of illustrating the changing proportion of false positives and true positives with varying risk thresholds, serves to complement deficiencies in the ROC curve.

### Statistical methods

For the statistical analysis, GraphPad Prism version 10.0 (GraphPad Software, MA, US) was utilized. The Chi-Square test was employed for analyzing nonparametric data, with results presented as median and quartile ranges. For parametric data, the Student’s *t*-test was used, with mean values and standard errors reported. A *p*-value of less than 0.05 was considered statistically significant in all analyses.

## CONCLUSIONS

The study highlights the importance of combining clinical indicators with CT and CTP parameters to predict the risk of hemorrhagic transformation after thrombectomy. This approach improves the accuracy of risk assessment, providing a more comprehensive tool for customizing treatment plans for each patient. It represents a notable step forward in the management of acute ischemic stroke, emphasizing the cooperative utilization of clinical findings and perfusion imaging for the purpose of patient outcome prediction.
